# Supplementary material for: Quantum transport protected by acceleration from nonadiabaticity and dissipation
Source: Nat Commun. 2025 Aug 20;16:7769. doi: 10.1038/s41467-025-62786-8 (PMC12368214; doi:10.1038/s41467-025-62786-8)
Supplement: Supplementary file 1 — Supplementary Information [file 41467_2025_62786_MOESM1_ESM.pdf]

Supplementary Information: Quantum Transport Protected by Acceleration From Nonadiabaticity and Dissipation

Arnab Chakrabarti[1,2*†], Biswarup Ash[3,4†], Igor Mazets[5,6], Xi Chen[7], Gershon Kurizki[1]

[1]AMOS and Department of Chemical and Biological Physics, Weizmann Institute of Science, Street, Rehovot, 7610001, Israel.
[2]Department of Physics, Rajiv Gandhi University, Rono Hills, Doimukh, 791112, Arunachal Pradesh, India.
[3]Department of Physics of Complex Systems, Weizmann Institute of Science, Rehovot, 7610001, Israel.
[4]Department of Physics, University of Michigan, Ann Arbor, 48109, MI, USA.
[5]Vienna Center for Quantum Science and Technology (VCQ), Atominstitut, TU Wien, Vienna, 1020, Austria.
[6]Wolfgang Pauli Institut c/o Fakultät für Mathematik, Universität Wien, Vienna, 1090, Austria.
[7]Instituto de Ciencia de Materiales de Madrid (CSIC), Cantoblanco, Madrid, E-28049, Spain.

*Corresponding author(s). E-mail(s): arnab.chakrabarti@rgu.ac.in;
†These authors contributed equally to this work.

I The Morse potential

In our numerical analysis, we focus on a moving Morse-trap where,

$$V[x - x_\circ(t)] = D\left[e^{-2a\{x-x_\circ(t)\}} - 2e^{-a\{x-x_\circ(t)\}}\right]. \tag{S1}$$

Here D denotes the depth of the potential, a is a parameter that assumes non-zero positive values determining the width of the Morse-trap, $x_\circ(t)$ is the time-dependent center of the well [s1].

II Instantaneous Eigenstates (eigen basis) of a wavepacket in a moving Morse potential

At each time-instant t the Hamiltonian $H_S(t)$ admits both bound (discrete) and continuous energy eigen-states [s2–s4] which are defined as the instantaneous energy eigenstates. The discrete sector, enumerated by integer quantum numbers n, has discrete eigen-frequencies given by [s2–s4]

$$\omega_n = -\frac{a^2}{2m}(N-n)^2, \tag{S2}$$

where n ranges from 0 to the integer part of N, which is given by

$$\left(N+\frac{1}{2}\right)^2 = \frac{2\,m\,D}{a^2}. \tag{S3}$$

The frequency dispersion relation for the continuous part is given by [s2–s4]

$$\omega_\kappa = \frac{a^2}{2m}\kappa^2\ ,\ \ \forall\,\kappa\in[0,\infty], \tag{S4}$$

where κ denotes the continuum index of the unbound impurity state. The corresponding discrete and continuous eigenfunctions, $\Phi[n, x-x_\circ(t)],\ \Phi[\kappa, x-x_\circ(t)]$ respectively, are given by

$$\Phi(n, z(t)) = \mathcal{N}_n\ [z(t)]^{N-n}\ e^{-z(t)/2}\ M[-n, 2N-2n+1, z(t)] \tag{S5}$$

and

$$\Phi(\kappa, z(t)) = \mathcal{N}(\kappa)\ [z(t)]^{-i\kappa}\ e^{-z(t)/2}\ U[-N-i\kappa, 1-2i\kappa, z(t)], \tag{S6}$$

where

$$z(t) = (2N+1)\,e^{-a[x-x_\circ(t)]}, \tag{S7}$$

$M(a,b,z)$ and $U(a,b,z)$ are Kummer functions of first and second kind respectively and

$$\mathcal{N}_n = \left[\frac{(2N-2n)\Gamma(2N-n+1)}{n!\Gamma(2N-n+1)^2}\right]^{\frac{1}{2}} \tag{S8}$$

while $\mathcal{N}(\kappa)$ is determined using $\langle\Phi[\kappa, z(t)]|\Phi[\kappa', z(t)]\rangle = \delta(\kappa-\kappa')$, as [s2–s4]

$$\mathcal{N}(\kappa) = \frac{|\Gamma(-N-i\kappa)|}{\pi}\sqrt{\kappa\sinh(2\pi\kappa)}. \tag{S9}$$

Both $\Phi[n, z(t)]$ and $\Phi[\kappa, z(t)]$ are real-valued functions of its arguments [s2–s4]. Motion of the trap-center $x_\circ(t)$ causes the time-variation of these eigenfunctions due to their dependence on $x-x_\circ(t)$. However, the instantaneous discrete and continuous eigenvalues are independent of the potential-center and hence of t.

We denote the single instantaneous bound-state in our problem by $|n(t)\rangle$ and the set of instantaneous continuum states by $\{|\kappa(t);\forall\kappa > 0\rangle\}$. Following [s5], we represent the integrals over the continuum modes as summations, for simplicity, but account for their continuous character whenever evaluation of the integrals is necessary.

In the instantaneous eigenbasis of the moving Morse-trap, the system (wavepacket) Hamiltonian can be written as

$$H_S(t) = \omega_\circ |n(t)\rangle\langle n(t)| + \sum_\kappa \omega_\kappa |\kappa(t)\rangle\langle\kappa(t)|. \tag{S10}$$

III Frölich coupling in the instantaneous eigenbasis

The system-bath interaction Hamiltonian H_{SB} has time-dependent matrix-elements in the instantaneous eigen basis of $H_S(t)$ and is of the general form:

$$\begin{aligned} H_{SB} &= \sum_k \left[b_{-k} \left\{ g_k\, e^{-ikx_\circ(t)} \right\} e^{-ik\{x - x_\circ(t)\}} + b^\dagger_{-k} \left\{ g_k\, e^{-ikx_\circ(t)} \right\}^* e^{ik\{x-x_\circ(t)\}} \right] \\ &= \sum_{k,\kappa} \left[b_{-k} \left\{ d^k_{n\kappa}(t)\, |n(t)\rangle\langle\kappa(t)| + d^k_{\kappa n}(t)\, |\kappa\rangle\langle n(t)| \right\} + h.c. \right] \\ &\quad + \sum_{k,\kappa,\epsilon} \left[b_{-k} \left\{ d^k_{\epsilon\kappa}(t)\, |\epsilon(t)\rangle\langle\kappa(t)| + d^k_{\kappa\epsilon}(t)\, |\kappa(t)\rangle\langle\epsilon(t)| \right\} + h.c. \right]. \end{aligned} \tag{S11}$$

Here, the phonon-induced transition matrix elements are defined as

$$d^k_{rs}(t) = \left\{ g_k\, e^{-ikx_\circ(t)} \right\} d^k_{rs}. \tag{S12}$$

with

$$d^k_{rs} = \langle r(t)|\, e^{-ik\{x-x_\circ(t)\}}\, |s(t)\rangle \tag{S13}$$

being time-independent.

In Eq. (S11), the indices κ, ϵ label instantaneous eigen-states in the continuum spectrum. The Hamiltonian (S11) includes both standard Rotating Wave Approximation-(RWA) terms as well as terms which do not comply with RWA (non-RWA). The non-RWA terms play significant role in short-time dynamics but become negligible in the long-time limit.

IV Trapped Wavepacket Dynamics

The state $|n(t)\rangle$ denotes the *instantaneous* bound eigenstate (eigenfunction) of the system Hamiltonian at time t, given by: $\Phi[n, z(t)]$ where $z(t) \propto e^{-a\{x-x_\circ(t)\}}$ and $x - x_\circ(t)$ is the time-dependent relative position of the trapped-particle with respect to the *instantaneous* trap center $x_\circ(t)$, as described in details in Eqs. (S5, S6, S7) (x is the absolute position of the trapped particle).

Now, the action of a single instance of the system-bath coupling (Frölich) Hamiltonian (see Eq. (2) of the manuscript) on the product state $|n(t)\rangle \otimes |0_{\rm bath}\rangle$ results in:

$$H_{SB}|n(t)\rangle \otimes |0_{\rm bath}\rangle = \sum_{k\neq 0} g^*_k\, e^{ikx}|n(t)\rangle \otimes |1^{-k}_{\rm bath}\rangle, \tag{S14}$$

where $|1_{\rm bath}^{-k}\rangle$ indicates the many-body bath state with a single-phonon excitation having momentum $-k$. The system state $e^{ikx}|n(t)\rangle = e^{ikx}\Phi[n, z(t)]$ can be expressed in the form

$$e^{ikx_\circ(t)}\phi[x - x_\circ(t)]. \tag{S15}$$

where $\phi[x-x_\circ(t)] = e^{ik\{x-x_\circ(t)\}}\Phi[n, z(t)]$ is a function of the relative coordinate $x-x_\circ(t)$. This implies that the wavepacket $e^{ikx}|n(t)\rangle$ has an additional momentum k associated with a fixed value of the trap-centre coordinate $x_\circ$, expressing the overall conservation of momentum (advanced/retarded wavepacket) [s6, s7]. On the other hand $|n(t)\rangle$ being the instantaneous eigenstate has no such momentum [s7]. This may also be understood by noting that the scattered wavepacket $e^{ikx}|n(t)\rangle = e^{ikx}\Phi[n, z(t)]$ is a gauge transformed eigenfunction of the trap, for which the transformed canonical momentum is shifted by k [s7, s8].

Alternatively, $e^{ikx}|n(t)\rangle$ may be thought of as an instantaneous eigenstate for a potential trap whose centre is moving with a speed $\dot{x}_\circ(t) + k/m$. To see this explicitly, we consider the coordinate transformation $q = x - x_\circ$, under which quantum mechanical wavefunctions transform as:

$$\psi(t, q = x - x_\circ) = e^{i\,m\,\left\{x\,\dot{x}_\circ - x_\circ\,\dot{x}_\circ + \frac{1}{2}\int_0^t\,\dot{x}_\circ^2\right\}}\psi(t, x), \tag{S16}$$

in order to satisfy the Schrödinger equation [s9, s10]. Omitting the time-dependent phase factors which are kixed at a particular instant t, the above equation can be simplified as:

$$\psi(t, q) = e^{i\,m\,x\,\dot{x}_\circ}\;\psi(t, x). \tag{S17}$$

Now for the scattered wavepacket $e^{ikx}|n(t)\rangle = e^{ikx}\Phi[n, z(t)]$, we invoke a coordinate transformation $q_1 = x + \frac{k}{m}t$ so that the wavepacket transforms to

$$e^{im(-\frac{k}{m})x}\;e^{ikx}\;\Phi[n, z(t)] := \Phi[n, z_1(t)] \tag{S18}$$

where $z_1(t) \propto e^{-a\{q_1-(\dot{x}_\circ+\frac{k}{m}t)\}}$. $\Phi[n, z_1(t)]$ is evidently the instantaneous eigenstate (upto an instantaneous global phase) for the potential $V[q_1 - (x_\circ + \frac{k}{m}t)]$ i.e. a potential moving with speed $\dot{x}_\circ + k/m$ (advanced/retarded trap). By similar arguments one can show that the states $\{e^{ikx}|\epsilon(t)\rangle\}$ describe eigenstates of advanced or retarded potential traps. Eq. (S14) then suggests that the action of the coupling H_{SB} on the initial state $|\psi(0)\rangle = |\nu(0)\rangle = |n(0)\rangle \otimes |0_{\rm bath}\rangle$, results in a many-body entangled state, made up of Fock-states with eigenstates of advanced or retarded traps moving with speed $\dot{x}_\circ + k/m$. Equivalently, H_{SB} couples instantaneous eigenfunctions with advanced or retarded wavepackets moving with speed k/m along with the creation of a phonon with momentum $-k$, that maintains the conservation of momentum in the many-body scattering process.

On the other hand, the non-adiabatic transitions connect instantaneous eigenstates, in traps moving with the same trap-speed, with no phonon-excitation and as such, conserve the overall momentum in the many-body dynamics.

Since the set of instantaneous eigenstates $\{|n(t)\rangle, |\epsilon(t)\rangle\ ;\ \forall\epsilon > 0\}$ form a complete basis for the system at any instant of time t and the bath states can be expanded in the Fock basis $\forall k \neq 0$, without loss of generality we expand the many-body (entangled) state at time $t > 0$

in a product basis (using Einstein's summation convention) as:

$$
\begin{aligned}
|\psi(t)\rangle = &\Big[\alpha_\circ(t)\, e^{-i\omega_\circ t}\, |n(t)\rangle \otimes |0_{\text{bath}}\rangle + \sum_k \alpha_{1k}(t)\, e^{-i(\omega_\circ+\Omega_k)t}\, |n(t)\rangle \otimes |1^{-k}_{\text{bath}}\rangle \\
&+ \sum_{k,k_1} \alpha_{2kk_1}(t)\, e^{-i(\omega_\circ+\Omega_k+\Omega_{k_1})t}\, |n(t)\rangle \otimes |2^{-(k+k_1)}_{\text{bath}}\rangle + ...\Big] \\
&+ \sum_\epsilon \Big[\beta_\epsilon(t)\, e^{-i\omega_\epsilon t}\, |\epsilon(t)\rangle \otimes |0_{\text{bath}}\rangle + \sum_k \beta_{\epsilon 1k}(t)\, e^{-i(\omega_\epsilon+\Omega_k)t}\, |\epsilon(t)\rangle \otimes |1^{-k}_{\text{bath}}\rangle \\
&+ \sum_{k,k_1} \beta_{\epsilon 2kk_1}(t)\, e^{-i(\omega_\epsilon+\Omega_k+\Omega_{k_1})t}\, |\epsilon(t)\rangle \otimes |2^{-(k+k_1)}_{\text{bath}}\rangle + ...\Big],
\end{aligned}
\tag{S19}
$$

where we have used the fact that Ω_k is symmetric in k (see Methods). The quantity within the first pair of square brackets on the r.h.s. of (S19) corresponds to the "bound" sector of the instantaneous system states in many-body product basis, while that inside the second pair of square brackets indicate the "unbound" sector. We note that the non-adiabatic transitions due to the motion of the trap cannot directly induce bath excitations.

V Wigner-Weisskopf Treatment

In order to make the calculation simple and transparent, we define infinite dimensional tensors: $\bar{\beta}_\epsilon$, $\bar{\beta}_{\epsilon 1k}$, $\bar{\beta}_{\epsilon 2kk_1}$,.... and $\bar{\alpha}_{1k}$, $\bar{\alpha}_{1kk_1}$, ... etc , where $0 \leq \epsilon < \infty$, $0 < k, k_1, \cdots < \infty$. Using these, we define higher-order (super)-tensors of the form

$$
\boldsymbol{B}(t) := \begin{bmatrix} \bar{\beta}_\epsilon \\ \bar{\beta}_{\epsilon 1k} \\ \bar{\beta}_{\epsilon 2kk_1} \\ \cdot \\ \cdot \\ \cdot \end{bmatrix} \quad ; \quad \boldsymbol{A}(t) := \begin{bmatrix} \alpha_\circ \\ \bar{\alpha}_{1k} \\ \bar{\alpha}_{2kk_1} \\ \cdot \\ \cdot \\ \cdot \end{bmatrix}
\tag{S20}
$$

in terms of which the Schrödinger equation can be expressed as:

$$
\begin{aligned}
\frac{d}{dt}\boldsymbol{A} &= -i\boldsymbol{F}^\dagger \boldsymbol{B} \\
\frac{d}{dt}\boldsymbol{B} &= \boldsymbol{M}\boldsymbol{B} - i\boldsymbol{F}\boldsymbol{A},
\end{aligned}
\tag{S21}
$$

where $\boldsymbol{M}$ and $-i\boldsymbol{F}^\dagger$ are super-tensor operators of the form :

$$\begin{bmatrix} -\tilde{\gamma}_{j\epsilon}(t) & -i\tilde{\mathbf{\Delta}}^{\boldsymbol{k}}_{j\epsilon}(t) & & \\ -i\tilde{\mathbf{\Delta}}^{\boldsymbol{k}\dagger}_{\epsilon j}(t) & -\tilde{\gamma}_{j\epsilon}(t) & -i\sqrt{2}\,\tilde{\mathbf{\Delta}}^{\boldsymbol{k_1}}_{j\epsilon}(t) & \\ 0 & -i\sqrt{2}\,\tilde{\mathbf{\Delta}}^{\boldsymbol{k_1}\dagger}_{\epsilon j}(t) & -\tilde{\gamma}_{j\epsilon}(t) & \\ & & \ddots & \ddots \;\ddots \end{bmatrix} \quad ; \quad j \in \{n, \{\epsilon > 0\}\}. \tag{S22}$$

The tensor products in equations (S21) use the standard convention where repeated indices are contracted (summed). In (S22), the operators $\tilde{\boldsymbol{\gamma}}_{j\epsilon}(t)$ denote phonon-number preserving, non-adiabatic transitions $\big[$either bound-to-continuum ($j = n$) in case of $-i\boldsymbol{F}^\dagger$ or continuum-to-continuum ($j \in \{\epsilon > 0\}$) in case of $\boldsymbol{M}\big]$ with matrix elements of the form

$$\gamma_{j\epsilon}(t) = \langle j(t)|\frac{\partial}{\partial t}|\epsilon(t)\rangle\, e^{-i\omega_{\epsilon j}t} = \frac{\langle j(t)|\frac{\partial H_S(t)}{\partial t}|\epsilon(t)\rangle}{\omega_{\epsilon j}}\, e^{-i\omega_{\epsilon j}t} = \dot{x}_\circ(t)\frac{\mu_{j\epsilon}}{\omega_{\epsilon j}}\, e^{-i\omega_{\epsilon j}t}, \tag{S23}$$

where, $\omega_{\epsilon j} = \omega_\epsilon - \omega_j$ and $\mu_{j\epsilon} = \int_{-\infty}^{\infty} dq\; \Phi[j,q]\Big[2\,a\,D\{e^{-2aq} - e^{-aq}\}\Big]\Phi[\epsilon,q]$ and we have used the variable substitution $q = x - x_\circ(t)$].

We note that (S23) cannot strictly describe continuum-continuum non-adiabatic transitions, since it may tend to diverge, having a vanishingly small denominator [s11]. This problem may be avoided by introducing a "virtual gap" in the continuous spectrum through re-defining the continuum eigenfunctions $\Phi(\kappa, z(t))$ as Weýl eigen-differential wave packets, which behave like discrete eigenfunctions [s12, s13]. One can then arrive at the limiting (finite) value of the non-adiabatic transition rates for a gapless spectrum [s12, s13]. However, these transitions are insignificant on the time-scales we wish to explore.

The operators $\tilde{\mathbf{\Delta}}^{\boldsymbol{k}}_{j\epsilon}(t)$ indicate transitions induced by the (de)excitation of a single phonon due to the Frölich coupling, with matrix elements

$$\Delta^k_{j\epsilon}(t) = d^k_{j\epsilon}(t)\, e^{-i\omega_{\epsilon j}t}\, e^{-i\Omega_k t}. \tag{S24}$$

We next perform the Wigner-Weisskopf calculation of the dynamics in two steps. First, we formally solve the second equation in (S21), to find

$$\boldsymbol{B}(t) = T\, e^{-\int_0^t ds \boldsymbol{M}(s)}\boldsymbol{B}(0) - i\int_0^t ds\; T\; e^{-\int_s^t d\tau \boldsymbol{M}(\tau)}\boldsymbol{F}(s)\;\boldsymbol{A}(s), \tag{S25}$$

where T denotes the chronological time-ordering operator. Since we assume that initially the wavepacket was trapped in the Morse potential, we must have $\boldsymbol{B}(0) = \mathbf{0}$. Then (S25) reduces to

$$\boldsymbol{B}(t) = -\,i\int_0^t ds\; \boldsymbol{U_M}(t,s)\;\boldsymbol{F}(s)\;\boldsymbol{A}(s), \tag{S26}$$

where we have defined $\boldsymbol{U_M}(t,s) = T\ \exp[-\int_s^t d\tau \boldsymbol{M}(\tau)]$. Substituting (S26) in the r.h.s. of the first equation in (S21), we then have

$$\frac{d}{dt}\boldsymbol{A}(t) = -\int_0^t ds \left[\boldsymbol{F}^\dagger(t)\boldsymbol{U_M}(t,s)\boldsymbol{F}(s)\right]\boldsymbol{A}(s). \tag{S27}$$

Thus, the effect of the unbound sector is formally integrated out as in the standard Wigner-Weisskopf treatment and (S27) describes the exact dynamics of the bound sector. In the lowest order of approximation, we replace $\boldsymbol{U_M}(t,s)$ by an identity operator and $\boldsymbol{A}(s)$ by $\boldsymbol{A}(t)$ in the kernel of (S27) to have

$$\frac{d}{dt}\boldsymbol{A}(t) = -\int_0^t ds\, \boldsymbol{F}^\dagger(t)\boldsymbol{F}(s)\,\boldsymbol{A}(t). \tag{S28}$$

We next find an equation for the probability amplitude $\alpha_\circ(t)$ from (S28) in the lowest order, by integrating out all other coefficients in the vector $\boldsymbol{A}(t)$ in the same manner as before, while using the initial condition that the bound-sector coefficients associated with excited phonon-states were identically zero at the beginning of the quench.

The rationale behind retaining only the lowest order contribution in the expressions for $\frac{d}{dt}\boldsymbol{A}(t)$ and hence $\frac{d}{dt}\alpha_\circ(t)$ is that the higher-order effects of systematic evolution become negligible upon averaging over a continuous and effectively infinite range of the bath or continuum frequencies [s14–s21]. This is true for couplings that are not strong enough to resolve the dynamics within the characteristic time-scales of this averaging. Since the transitions governed by $\boldsymbol{U_M}$ in (S27) spread the wave packet throughout the continuum, we can estimate that effects beyond the leading order would be observable within a time-scale of the order of the inverse of the continuum width (energy uncertainty [s22]), which can be much smaller than the measurement resolution. For phonon-induced transitions, this characteristic time can be estimated as $\tau_B = (r_B/\xi)^2\, t_B$ where r_B denotes the charachteristic length-scale of inter-atomic interactions, t_B being the coherence time of impurity dynamics in BEC as before [s19]. Since r_B can be orders of magnitude smaller than the bath coherence length ξ, we have $\tau_B \ll t_B$ [s19]. Both non-adiabatic and phonon-mediated coupling strengths explored here are weaker than the inverse of the characteristic times discussed above so that our theory is accurate for times $t > \max\{\tau_B\,,\ \Delta\omega^{-1}\}$, $\Delta\omega$ being the continuum width.

The resulting dynamical equation for the amplitude $\alpha_\circ(t)$ [defined in (S19)], after this two-step Wigner-Weisskopf protocol, while retaining only the lowest order terms in the trap-speed and system-bath coupling (see Eq. (S28)) has the form:

$$\frac{d\alpha_\circ}{dt} = -\int_0^t ds\ \Sigma(t,s)\alpha_\circ(t), \tag{S29}$$

where the kernel (self-energy) is given by, $\Sigma(t,s) = \left\{\gamma_{n\epsilon}(t)\,\gamma^*_{n\epsilon}(s) + \Delta^k_{n\epsilon}(t)\,\Delta^{k*}_{n\epsilon}(s)\right\}$ (repeated indices are summed). With the initial condition $\alpha_\circ(0) = 1$ we then solve the above equation exactly, to have

$$
\begin{aligned}
\alpha_\circ(t) &= \exp\Big[-\int_0^t dt_1 \int_0^{t_1} dt_2\ \Sigma(t_1,t_2)\Big] \\
&= 1 - \int_0^t dt_1 \int_0^{t_1} dt_2 \left\{ \gamma_{n\epsilon}(t_1)\,\gamma^*_{n\epsilon}(t_2) + \Delta^k_{n\epsilon}(t_1)\,\Delta^{k*}_{n\epsilon}(t_2) \right\} \\
&\quad + \int_0^t dt_1 \int_0^{t_1} dt_2 \int_0^{t_2} dt_3 \int_0^{t_3} dt_4 \left\{ \gamma_{n\epsilon}(t_1)\,\gamma^*_{n\epsilon}(t_3) + \Delta^k_{n\epsilon}(t_1)\,\Delta^{k*}_{n\epsilon}(t_3) \right\} \\
&\qquad\qquad \left\{ \gamma_{n\epsilon}(t_2)\,\gamma^*_{n\epsilon}(t_4) + \Delta^k_{n\epsilon}(t_2)\,\Delta^{k*}_{n\epsilon}(t_4) \right\} \\
&\quad + \quad \ldots\ldots\ldots, \qquad \text{(S30)}
\end{aligned}
$$

where in the last-step we have used the fact that $\Sigma(t_1, t_2)$ is symmetric in t_1 and t_2.

Since $\Sigma(t_1, t_2)$ is quadratic in $\dot{x}_\circ$ and H_{SB}, the above expression is a re-summation of all second-order diagrams akin to a Dyson series [s23, s24],. This Wigner-Weisskopf non-perturbative approach is widely used in quantum optics [s23–s27]. Hence, the survival probability $|\alpha_\circ(t_f)|^2$ in (S31) goes beyond linear response, since it takes into account the cumulative effect of an infinite sequence of second-order self-energies.

VI Dynamical Fidelity and Loschmidt echo

From the discussion in Sec. (IV) and Methods A of the manuscript, it follows that a non-zero phonon excitation in the general state corresponds to advanced or retarded wavepackets. Thus fidelity (survival probability), defined as the transition probability between two pure quantum states [s28, s29] $\big[$in our case : perturbed state $|\psi(t)\rangle$ and unperturbed state $|n(t)\rangle \otimes |0_{\rm bath}\rangle\big]$ is given by $|\alpha_\circ(t)|^2$, i.e. the probability of finding the many-body state in $|n(t)\rangle \otimes |0_{\rm bath}\rangle$ (Loschmidt-echo). All other many-body states having non-zero phonon excitations, corresponding to advanced or retarded wavepacket states, are orthogonal to the state $|n(t)\rangle \otimes |0_{\rm bath}\rangle$ so that projecting on to $|n(t)\rangle \otimes |0_{\rm bath}\rangle$ we eliminate all other phonon states that do not contribute to the fidelity.

The reduced density matrix $\rho_S(t) = {\rm Tr}_{\rm bath}\big(|\psi(t)\rangle\langle\psi(t)|\big)$ is a mixed state composed of advanced or retarded (gauge-transformed) and instantaneous system eigenstates, since $|\psi(t)\rangle$ is an entangled system-bath state [Sec. (IV)]. For mixed states (density matrices) [s29, s30], the most common fidelity measure is the Uhlmann-Josza fidelity $F(\rho_S(t), \sigma) = \Big({\rm Tr}\sqrt{\sqrt{\rho_S(t)}\sigma\sqrt{\rho_S(t)}}\Big)^2$, including its version when one of the density matrices is pure (Schumacher's fidelity: $\langle\psi|\rho|\psi\rangle$), defined as the maximal transition probability between purifications of the two density matrices [s29–s31]. Yet such a fidelity measure relies on the assumption that the two density matrices being compared, are derived from identically enlarged Hilbert spaces, which is not always the case [s29]. Moreover, being the maximum over purifications, such a fidelity measure over a reduced Hilbert space may be greater than

the relevant fidelities for two pure states [s29]. In our problem, the instantaneous and scattered wavepackets $|n(t)\rangle$ and $e^{ikx}|n(t)\rangle$ may have a non-zero overlap in general, due to their finite width. However, the bath states $|0_{\rm bath}\rangle$, $|1^{-k}_{\rm bath}\rangle$ are all orthogonal, hence the many body states $e^{ikx}|n(t)\rangle \otimes |1^{-k}_{\rm bath}\rangle$ and $|n(t)\rangle \otimes |0_{\rm bath}\rangle$ are orthogonal. Therefore, tracing out the bath degrees of freedom reduces the distinguishability between scattered and unscattered contributions to the instantaneous states $|n(t)\rangle$. As a result, the corresponding Schumacher's fidelity $\langle n(t)|\rho_S(t)|n(t)\rangle$ may include contributions from the scattered (advanced/retarded states) and exceed the true survival probability in the instantaneous state $|n(t)\rangle$, required for quantum state preservation."

Note that the set of states $\mathcal{B} := \{|n(t)\rangle \otimes |1^{-k}_{\rm bath}\rangle, |n(t)\rangle \otimes |2^{-(k+k_1)}_{\rm bath}\rangle...., |\epsilon(t)\rangle \otimes |0_{\rm bath}\rangle, |\epsilon(t)\rangle \otimes |1^{-k}_{\rm bath}\rangle, |\epsilon(t)\rangle \otimes |2^{-(k+k_1)}_{\rm bath}\rangle... \quad \forall \epsilon > 0, k \neq 0\}$ defines a hyper-surface in the system-bath Fock space, orthogonal to the instantaneous state $|n(t)\rangle \otimes |0_{\rm bath}\rangle$. On the other hand, the instantaneous scattered states $\{e^{ikx}|n(t)\rangle \otimes |1^{-k}_{\rm bath}\rangle, e^{ikx}|n(t)\rangle \otimes |2^{-(k+k_1)}_{\rm bath}\rangle....e^{ikx}|n(t)\rangle \otimes |1^{-k}_{\rm bath}\rangle, e^{ikx}|n(t)\rangle \otimes |2^{-(k+k_1)}_{\rm bath}\rangle....\}$ are also orthogonal to $|n(t)\rangle \otimes |0_{\rm bath}\rangle$ and hence lie entierly on the hyper-surface $\mathcal{B}$.

So from Eq. (S30) of the SI, the survival probability (Loschmidt echo) at $t = t_f$, is:

$$\mathcal{P}(t_f) = \left|\alpha_\circ(t_f)\right|^2 = \exp\left[-2\ {\rm Re} \int_0^{t_f} dt_1 \int_0^{t_1} dt_2 \sum_{k,\epsilon} \Big\{ \gamma_{n\epsilon}(t_1)\, \gamma^*_{n\epsilon}(t_2) + \Delta^k_{n\epsilon}(t_1)\, \Delta^{k*}_{n\epsilon}(t_2) \Big\} \right], \tag{S31}$$

The first term in the integrand of equation (S31) accounts for the non-adiabatic contribution to the loss of fidelity while the second term indicates phonon-mediated contributions to the same, arising from the non-RWA terms in (S11). Using,

$$\text{Re}\big[\gamma_{n\epsilon}(t_1)\, \gamma^*_{n\epsilon}(t_2)\big] = \dot{x}_\circ(t_1)\dot{x}_\circ(t_2) \sum_\epsilon \frac{|\mu_{n\epsilon}|^2}{\omega^2_{\epsilon n}} \cos\big[\omega_{\epsilon n}(t_1 - t_2)\big], \tag{S32}$$

and

$$\text{Re}\big[\Delta^k_{n\epsilon}(t_1)\, \Delta^{k*}_{n\epsilon}(t_2)\big] = \sum_{k\epsilon} |g_k d^k_{n\epsilon}|^2 \cos\big[(\omega_{\epsilon n} + \Omega_k)(t_1 - t_2) + k\{x_\circ(t_1) - x_\circ(t_2)\}\big] \tag{S33}$$

are symmetric in the dummy variables t_1 , t_2 and thus we can rewrite the nested integral in (S31) as a double integral to get

$$\mathcal{P}(t) = \exp\Big(- J[x_\circ, \dot{x}_\circ]\Big), \tag{S34}$$

where

$$J[x_\circ, \dot{x}_\circ] = \int_0^{t_f} dt_1 \int_0^{t_f} dt_2\ \dot{x}_\circ(t_1)\, \dot{x}_\circ(t_2) \sum_\epsilon \frac{|\mu_{n\epsilon}|^2}{\omega^2_{\epsilon n}} \cos\Big[\omega_{\epsilon n}(t_1 - t_2)\Big]$$

$$+\int_0^{t_f} dt_1 \int_0^{t_f} dt_2 \sum_{k,\epsilon} |g_k\, d^k_{n\epsilon}|^2 \cos\Big[(\omega_{\epsilon n}+\Omega_k)(t_1-t_2) + k\big\{x_\circ(t_1)-x_\circ(t_2)\big\}\Big]. \tag{S35}$$

VII Non-local Euler-Lagrange (EL) equation

Since $J[x_\circ, \dot{x}_\circ]$ has the form of a double integral, we can calculate the first variation δJ as outlined in [s32]. To this end, using the identity $\cos(A-B) = \cos(A)\cos(B) + \sin(A)\sin(B)$, we rewrite equation (S35) as

$$J[x_\circ, \dot{x}_\circ] = \int_0^{t_f} dt_1 L_0\big[t_1, \dot{x}_\circ(t_1), K_0[t_1, \dot{x}_\circ]\big] + \sum_{k,\epsilon} \int_0^{t_f} dt_1 L_1\big[t_1, x_\circ(t_1), K_1[x_\circ]\big] + \sum_{k,\epsilon} \int_0^{t_f} dt_1 L_2\big[t_1, x_\circ(t_1), K_2[x_\circ]\big]. \tag{S36}$$

Here

$$L_0\big[t_1, \dot{x}_\circ(t_1), K_0[t_1, \dot{x}_\circ]\big] = \dot{x}_\circ(t_1) K_0[t_1, \dot{x}_\circ], \tag{S37}$$

$$K_0[t_1, \dot{x}_\circ] = \int_0^{t_f} dt_2\, \dot{x}_\circ(t_2) \sum_{\epsilon} \frac{|\mu_{n\epsilon}|^2}{\omega^2_{\epsilon n}} \cos\Big[\omega_{\epsilon n}(t_1-t_2)\Big], \tag{S38}$$

$$L_1\big[t_1, x_\circ(t_1), K_1[x_\circ]\big] = |g_k\, d^k_{n\epsilon}|^2 \cos\Big[(\omega_{\epsilon n}+\Omega_k)t_1 + k x_\circ(t_1)\Big] K_1[x_\circ], \tag{S39}$$

$$K_1[x_\circ] = \int_0^{t_f} dt_2 \cos\Big[(\omega_{\epsilon n}+\Omega_k)t_2 + k x_\circ(t_2)\Big] \tag{S40}$$

$$L_2\big[t_1, x_\circ(t_1), K_2[x_\circ]\big] = |g_k\, d^k_{n\epsilon}|^2 \sin\Big[(\omega_{\epsilon n}+\Omega_k)t_1 + k x_\circ(t_1)\Big] K_2[x_\circ] \tag{S41}$$

and

$$K_2[x_\circ] = \int_0^{t_f} dt_2 \sin\Big[(\omega_{\epsilon n}+\Omega_k)t_2 + k x_\circ(t_2)\Big]. \tag{S42}$$

Thus,

$$\delta J[x_\circ, \dot{x}_\circ] = \int_0^{t_f} dt_1 \Big[\frac{\partial L_0}{\partial \dot{x}_\circ(t_1)} \delta \dot{x}_\circ(t_1) + \frac{\partial L_0}{\partial K_0} \delta K_0\Big] + \sum_{k,\epsilon} \int_0^{t_f} dt_1 \Big[\frac{\partial L_1}{\partial x_\circ(t_1)} \delta x_\circ(t_1) + \frac{\partial L_1}{\partial K_1} \delta K_1\Big]$$

$$+\sum_{k,\epsilon}\int_0^{t_f} dt_1\left[\frac{\partial L_2}{\partial x_\circ(t_1)}\delta x_\circ(t_1)+\frac{\partial L_2}{\partial K_2}\delta K_2\right]. \tag{S43}$$

We evaluate

$$\begin{aligned}\int_0^{t_f} dt_1\,\frac{\partial L_0}{\partial \dot{x}_\circ(t_1)}\delta\dot{x}_\circ(t_1) &= -\int_0^{t_f} dt_1\,\frac{d}{dt_1}\left[\frac{\partial L_0}{\partial \dot{x}_\circ(t_1)}\right]\delta x_\circ(t_1)\\ &= \int_0^{t_f} dt_1\int_0^{t_f} dt_2\;\dot{x}_\circ(t_2)\sum_\epsilon \frac{|\mu_{n\epsilon}|^2}{\omega_{\epsilon n}}\sin\Big[\omega_{\epsilon n}(t_1-t_2)\Big]\delta x_\circ(t_1)\end{aligned} \tag{S44}$$

and

$$\int_0^{t_f} dt_1\,\frac{\partial L_0}{\partial K_0}\delta K_0 = -\int_0^{t_f} dt_1\int_0^{t_f} dt_2\,\sum_\epsilon \frac{|\mu_{n\epsilon}|^2}{\omega_{\epsilon n}}\dot{x}_\circ(t_1)\sin\Big[\omega_{\epsilon n}(t_1-t_2)\Big]\delta x_\circ(t_2). \tag{S45}$$

Interchanging the dummy variables t_1 and t_2 in the r.h.s. of (S45) equation we have

$$\begin{aligned}\int_0^{t_f} dt_1\,\frac{\partial L_0}{\partial K_0}\delta K_0 &= -\int_0^{t_f} dt_2\int_0^{t_f} dt_1\,\sum_\epsilon \frac{|\mu_{n\epsilon}|^2}{\omega_{\epsilon n}}\dot{x}_\circ(t_2)\sin\Big[\omega_{\epsilon n}(t_2-t_1)\Big]\delta x_\circ(t_1)\\ &= \int_0^{t_f} dt_1\int_0^{t_f} dt_2\;\dot{x}_\circ(t_2)\sum_\epsilon \frac{|\mu_{n\epsilon}|^2}{\omega_{\epsilon n}}\sin\Big[\omega_{\epsilon n}(t_1-t_2)\Big]\delta x_\circ(t_1).\end{aligned} \tag{S46}$$

So,

$$\begin{aligned}&\int_0^{t_f} dt_1\left[\frac{\partial L_0}{\partial \dot{x}_\circ(t_1)}\delta\dot{x}_\circ(t_1)+\frac{\partial L_0}{\partial K_0}\delta K_0\right]\\ &\qquad = 2\int_0^{t_f}\left[dt_1\int_0^{t_f} dt_2\;\dot{x}_\circ(t_2)\sum_\epsilon \frac{|\mu_{n\epsilon}|^2}{\omega_{\epsilon n}}\sin\Big\{\omega_{\epsilon n}(t_1-t_2)\Big\}\right]\delta x_\circ(t_1).\end{aligned} \tag{S47}$$

Similarly we have,

$$\sum_{k,\epsilon} \int_0^{t_f} dt_1 \Big[\frac{\partial L_1}{\partial x_\circ(t_1)}\delta x_\circ(t_1) + \frac{\partial L_1}{\partial K_1}\delta K_1\Big]$$
$$= -2\int_0^{t_f} dt_1 \left[\sum_{k\epsilon} k|g_k d^k_{n\epsilon}|^2 \int_0^{t_f} dt_2 \sin\Big\{(\omega_{\epsilon n}+\Omega_k)t_1 + kx_\circ(t_1)\Big\}\right.$$
$$\left.\cos\Big\{(\omega_{\epsilon n}+\Omega_k)t_2 + kx_\circ(t_2)\Big\}\right]\delta x_\circ(t_1) \tag{S48}$$

and

$$\sum_{k,\epsilon} \int_0^{t_f} dt_1 \Big[\frac{\partial L_2}{\partial x_\circ(t_1)}\delta x_\circ(t_1) + \frac{\partial L_2}{\partial K_2}\delta K_2\Big]$$
$$= 2\int_0^{t_f} dt_1 \left[\sum_{k\epsilon} k|g_k d^k_{n\epsilon}|^2 \int_0^{t_f} dt_2 \cos\Big\{(\omega_{\epsilon n}+\Omega_k)t_1 + kx_\circ(t_1)\Big\}\right.$$
$$\left.\sin\Big\{(\omega_{\epsilon n}+\Omega_k)t_2 + kx_\circ(t_2)\Big\}\right]\delta x_\circ(t_1). \tag{S49}$$

The first variation of the constraint functional $J_1[\dot{x}_\circ]$ gives

$$\lambda\delta J_1[\dot{x}_\circ] = -2\lambda\int_0^{t_f} dt_1\big[\ddot{x}_\circ(t_1)\big]\delta x_\circ(t_1). \tag{S50}$$

Using (S43), (S47), (S48), (S49), (S50) and the identity: $\sin(A-B) = \sin(A)\cos(B)$ $-\cos(A)\sin(B)$ we arrive at the following form of $\delta J_{\rm tot}[x_\circ, \dot{x}_\circ]$:

$$\delta J_{\rm tot}[x_\circ, \dot{x}_\circ] = 2\int_0^{t_f} dt_1\left[\int_0^{t_f} dt_2\ \dot{x}_\circ(t_2)\sum_\epsilon \frac{|\mu_{n\epsilon}|^2}{\omega_{\epsilon n}}\sin\Big\{\omega_{\epsilon n}(t_1-t_2)\Big\}\right.$$
$$-\int_0^{t_f} dt_2 \sum_{k\epsilon} k|g_k d^k_{n\epsilon}|^2 \sin\Big\{(\omega_{\epsilon n}+\Omega_k)(t_1-t_2) + k\{x_\circ(t_1)-x_\circ(t_2)\}\Big\}$$
$$\left.-\lambda\ddot{x}_\circ(t_1)\right]\delta x_\circ(t_1). \tag{S51}$$

The *non-local* Euler-Lagrange (EL) equation is then obtained from the condition:

$$\delta J_{\rm tot}[x_\circ, \dot{x}_\circ] = 0. \tag{S52}$$

VIII Bound-to-continuum non-adiabatic transitions

Since we have a single bound state in our problem, the quantum number $n = 0$ in our case. For explicit evaluation of the optimal trajectory we need to calculate the squared transition matrix element $|\mu_{0\epsilon}|^2$. From the definition of $\mu_{n\epsilon}$ [see (S23)] we have

$$\mu_{0\epsilon} = 2aD \int_0^\infty \frac{dz}{a\,z}\,\Phi(0,z)\Big[\Big\{\frac{z}{(2N+1)}\Big\}^2 - \frac{z}{(2N+1)}\Big]\Phi(\epsilon,z) \tag{S53}$$

where, equation (S7), we have used the variable substitution $z = (2N+1)\,e^{-aq}$. Simplifying, we have

$$\begin{aligned}\mu_{0\kappa} &= \frac{2D}{(2N+1)^2}\int_0^\infty dz\;\phi_0(z)\;z\;\phi_\kappa(z)\\ &\quad -\frac{2D}{(2N+1)}\int_0^\infty dz\;\phi_0(z)\;\phi_\kappa(z)\\ &:= \frac{2D}{(2N+1)^2}\,I_1 - \frac{2D}{(2N+1)}\,I_2,\end{aligned} \tag{S54}$$

where in the last step we have denoted the first and second integrals on the r.h.s. as I_1 and I_2. Using (S5 – S7) and expressing the Kummer functions of the second-kind, $U(a,b,z)$, in terms of the Whittaker's function $W_{\lambda\mu}(z)$ [s33] we can evaluate I_1 and I_2 [s34], as

$$I_1 = \mathcal{N}_0\mathcal{N}(\kappa)\;\Gamma(N+2+i\kappa)\Gamma(N+2-i\kappa). \tag{S55}$$

and

$$I_2 = \mathcal{N}_0\mathcal{N}(\kappa)\;\Gamma(N+1+i\kappa)\Gamma(N+1-i\kappa). \tag{S56}$$

Substituting (S55) and (S56) on the r.h.s. of (S54) we obtain,

$$\begin{aligned}\mu_{0\kappa} = \frac{2D\mathcal{N}_0\mathcal{N}(\kappa)}{(2N+1)^2}\Big[\;&\Gamma(N+2+i\kappa)\;\Gamma(N+2-i\kappa)\\ &-(2N+1)\;\Gamma(N+1+i\kappa)\;\Gamma(N+1-i\kappa)\;\Big].\end{aligned} \tag{S57}$$

IX Bound-to-continuum bath-mediated transition:

The bound-to-continuum phonon-mediated transition matrix element $d^k_{n\epsilon}$ can be calculated as [s2]:

$$
\begin{aligned}
d^k_{n\epsilon} &= \langle n(t)|e^{-ikq}|\epsilon(t)\rangle \\
&= \langle \epsilon(t)|e^{ikq}|k(t)\rangle^* \\
&= \Big[A_n(\epsilon,k)\,{}_3F_2(-n, N-n+i\epsilon-ik, N-n-i\epsilon-ik; -n-ik, 2N-2n+1; 1)\Big]^*,
\end{aligned}
\tag{S58}
$$

where

$$
\begin{aligned}
A_n(\epsilon,k) &= \sqrt{\frac{(2N-2n)\Gamma(2N-n+1)\epsilon\sinh(2\pi\epsilon)}{n!}} \\
&\quad\times\frac{(2N+1)^{ik}(-1)^n\sinh(\pi k)|\Gamma(-N+i\epsilon)|}{i\pi^2\Gamma(2N-2n+1)} \\
&\quad\times\Gamma(1+n+ik)\Gamma(N-n-i\epsilon-ik)\Gamma(N-n+i\epsilon-ik)
\end{aligned}
\tag{S59}
$$

and ${}_3F_2$ is the generalized hypergeometric function [s2]. In our problem $n = 0$, which gives ${}_3F_2(0, N+i\epsilon-ik, N-i\epsilon-ik; -ik, 2N+1; 1) = 1$ and thus $d^k_{n=0\epsilon} = A^*_{n=0}(\epsilon,k)$. The dipole transition matrix element $D_{n\epsilon}$ is [s2]:

$$
D_{n\epsilon} = D^*_{\epsilon n} \tag{S60}
$$

$$
D_{\epsilon n} = \frac{(-1)^{n+1}|\Gamma(-N+i\epsilon)|}{\pi[(N-n)^2+\epsilon^2]}\sqrt{\frac{\epsilon\sinh(2\pi\epsilon)(2N-2n)}{n!\Gamma(2N-n+1)}}|\Gamma(1+N+i\epsilon)|^2. \tag{S61}
$$

X Solution of the Boundary Value Problem using Green's Function

The Euler-Lagrange (EL) equation, Eq. (12) together with Eq. (13), is a second-order integro-differential equation, which admits two arbitrary constants, specified by the two BCs: $x_\circ(0) = \text{constant}$ and $x_\circ(t_f) = \text{constant}$. In order to arrive at the simplified version Eq. (8) we have applied an additional time-derivative on both sides of Eq. (12). This comes with a penalty of requiring a third BC: Eq. (8) is second order in $v(t)$ and thus admits two independent BCs of $v(t)$. Together with the equation

$$
\dot{x}_\circ(t) = v(t), \tag{S62}
$$

we then have three independent BCs in the solution for the optimal trajectory $x_\circ(t)$. Upon fixing the initial speed, $v(0)$, in addition to $x_\circ(0)$ and $x_\circ(t_f)$, we then uniquely specify the optimal trajectory including the final trap-speed $v(t_f) = \dot{x}_\circ(t_f)$.

On the other hand, for comparison with CDF, the useful BCs are to set the initial and final trap speeds to be 0 i.e. we choose $v(0) = v(t_f) = 0$ [s7]. Fixing the initial trap position $x_\circ(0)$ ($= 0$ in our case), we then uniquely determine the optimal trap-trajectory from Eq. (8) and (S62), which inevitably fixes the final trap-position $x_\circ(t_f)$. One may instead choose to fix the initial and final trap positions along with the choice of $v(0) = 0$, which would then fix $v(t_f)$.

In order to show the superiority of our method over CDF, we have chosen the fixed final trap speed to be non-zero, a case where CDF fails but our method works well.

For transport without a bath, $v(t)$ serves as the generalized coordinate for the EL optimization (see Eq. (9)). In this case, the BCs fix $v(0) = \text{constant}$ and $v(t_f) = \text{constant}$ instead of $x_\circ(0)$ and $x_\circ(t_f)$. Integrating over the optimal velocity profile we then get the optimal trajectory, which is uniquely determined by the initial position $x_\circ(0)$, which in turn fixes $x_\circ(t_f)$ as before. The corresponding survival probabilities are plotted in Fig. 3D. In this case, the control problem becomes solvable only after introducing the additional constraint on the total distance covered by the trap-center, $J_2[v]$, which fixes $x_\circ(t_f)$ while $x_\circ(0)$ is set by the BC.

To obtain the optimal trajectory $x_\circ(t)$, we choose to solve the boundary value problem (BVP):

$$\lambda\,\ddot{v}(t) = -\eta(t) - \zeta(t)\,v(t) + \int_0^{t_f} ds\,\phi(t-s)\,v(s) := \mathcal{M}(t), \tag{S63}$$

$$v(0) = 0 \qquad ; \qquad v(t_f) = 0. \tag{S64}$$

along with (S62) with the initial condition $x_\circ(0) = 0$. First, we reduce the BVP (S63) & (S64), to a Fredholm integral equation [s35, s36] using the Green's function for the problem

$$\lambda\,\ddot{v}(t) = \mathcal{M}(t), \tag{S65}$$

$$v(0) = 0 \qquad ; \qquad v(t_f) = 0. \tag{S66}$$

given by [s37]

$$G(t,s) = \begin{cases} -\frac{1}{\lambda\,t_f}\,t\,(t_f - s) & 0 \le t < s \\ \\ -\frac{1}{\lambda\,t_f}\,s\,(t_f - t) & s < t \le t_f. \end{cases} \tag{S67}$$

Then the solution to the BVP (S65) & (S66) is given by

$$v(t) = \int_0^{t_f} ds\,G(t,s)\,\mathcal{M}(s). \tag{S68}$$

Using the definition of $\mathcal{M}(t)$ given in (S63) we then have to solve the Fredholm integral equation of the second kind:

$$v(t) = \mathcal{F}(t) + \int_0^{t_f} ds_1\,K(t,s_1)v(s_1), \tag{S69}$$

where

$$\mathcal{F}(t) = -\int_0^{t_f} ds\,G(t,s)\,\eta(s), \tag{S70}$$

$$K(t, s_1) = \mathcal{H}(t, s_1) - G_1(t, s_1), \tag{S71}$$
$$G_1(t, s_1) = G(t, s_1)\,\zeta(s_1) \tag{S72}$$

and

$$\mathcal{H}(t, s_1) = \int_0^{t_f} ds\, G(t, s)\,\phi(s - s_1). \tag{S73}$$

Thus, using the Green's function we have reduced the BVP (S63) & (S64) to a Fredholm integral equation (S69). This integral equation can be solved using the Liouville-Neumann series as

$$v(t) = \lim_{n\to\infty} \sum_{j=0}^{n} v^{(j)}(t), \tag{S74}$$

where

$$\begin{aligned} v^{(0)}(t) &= \mathcal{F}(t), \\ v^{(j)}(t) &= \int_0^{t_f} ds_1\, K(t, s_1) v^{(j-1)}(s_1). \end{aligned} \tag{S75}$$

Integrating (S74) over t, with the initial condition $x_\circ(0) = 0$, we obtain the optimal trajectory $x_\circ(t)$.

We use a modified Green's Function when the BCs are $v(0) = 0$, $v(t_f) = c_1 \neq 0$. In this case, we rewrite the (S63) in terms of a new variable

$$Q(t) = v(t) - c_1 \frac{t}{t_f}. \tag{S76}$$

(S63) then becomes

$$\lambda\, \ddot{Q}(t) = -\eta(t) - \zeta(t)\left(Q(t) + c_1 \frac{t}{t_f}\right) + \int_0^{t_f} ds\, \phi(t - s)\left(Q(s) + c_1 \frac{s}{t_f}\right). \tag{S77}$$

Here, $Q(t)$ satisfies the homogeneous boundary conditions $Q(0) = 0$ and $Q(t_f) = 0$.We solve this equation following the same method as for (S63) and (S64).

XI Numerical method to compute survival probability

Our main objective is to compute the survival probability $\mathcal{P}(t)$ at different times t for different parameters using (S34) and (S35) and hence to compute $v(t)$ and $x_\circ(t)$ which are required to calculate $\mathcal{P}(t)$. To comptute $v(t)$ we use the series solution method outlined in (S74). Namely, we numerically evaluate the r.h.s. of (S69) using the Green's function given in (S67) and hence find the Liouville-Neumann series solution of (S69) as described in (S74).

From (S74) and (S75), the solution for $v(t)$ depends on the number of terms j, being a series solution. Thus, we first determine the optimal j required to obtian $v(t) = \dot{x}_\circ(t)$. As

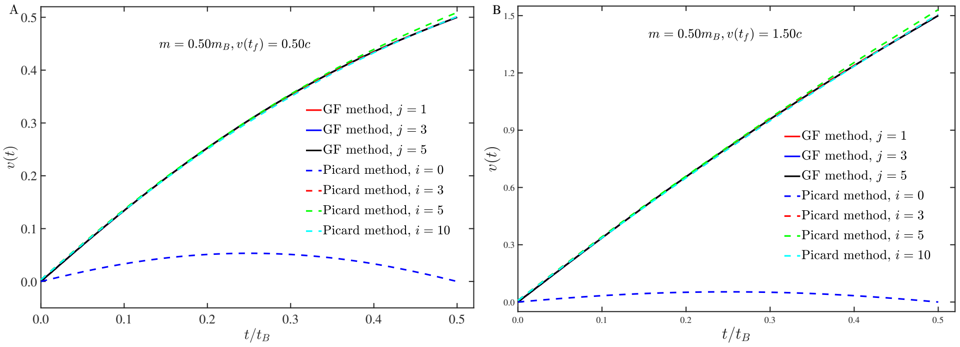

Supplementary Fig. S-1 Comparison of the approximate and exact numerical solution for the optimal trap-trajectory: Convergence of the trap-velocity profile obtained from the Green's function (GF) method and the Picard method with iteration number j and i, respectively. Note that the convergence to a stable velocity profile is achieved much faster in case of the GF method. For $j > 3$ and $i > 5$ both the methods yield same time dependence of the optimal trap-velocity $v(t)$. The convergence of the velocity profile $v(t)$ in GF and Picard methods are illustrated for two different values of the final velocities: **A** $v(t_f) = 0.5\,c$ and **B** $v(t_f) = 1.5\,c$ with $m = 0.5\,m_B$.

shown in Fig. S-1, for $\lambda = 1, m = 0.5\,m_B$, and $a = 1$ we find that the solution converges rapidly for $j \geq 3$. Such rapid convergence occurs also for other sets of parameters used here. We thus set $j = 50$ for all the parameters used, in order to ensure a stable solution for $v(t)$. Having obtained $v(t)$, we numerically integrate it to obtain $x_\circ(t)$. With this optimal solution for $v(t)$ and $x_\circ(t)$ we numerically evaluate the r.h.s. of S35 to obtain $J[x_\circ, \dot{x}_\circ]$, which yields the survival probability $\mathcal{P}(t)$. Note that, using the above procedure we have calculated the optimal trajectories both in case of $v(t_f) = 0$ and $v(t_f) = c_1 \neq 0$. In the latter case we perform the transformations (S76) and (S77) before applying the numerical protocol described above. Once the optimal trajectory is obtained under this transformed scheme, we easily obtain the actual optimal trajectory by applying a back transformation of the form $v(t) = Q(t) + c_1 \frac{t}{t_f}$ and use it to calculate $\mathcal{P}(t_f)$ as before. The corresponding numerical results are shown in the main text. For $v(t_f) = 0$ we directly solve (S69) using our numerical protocol to obtain the optimal trajectory and hence $\mathcal{P}(t_f)$ as shown in Fig. S-2.

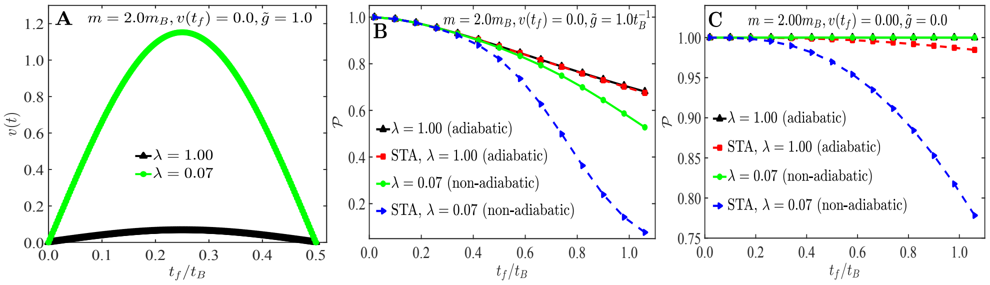

Supplementary Fig. S-2 Optimal transport with $v(t_f) = 0$: **A** Optimal trap speed for $m = 2.0\,m_B$, $\tilde{g} = 1.0t_B^{-1}$ and all other parameters as given in Methods C of the main text, with $\lambda = 1$ (black) and $\lambda = 0.07$ (green) **B** Corresponding survival probabilities. **C** Survival probabilities for non-adiabatic transport through vacuum ($\tilde{g} = 0$).

The BCs $v(0) = 0$ and $v(t_f) = 0$ yields an adiabatic optimal trajectory for $\lambda = 1$ with our chosen set of parameters, as shown in Fig. S-2 A (black curve). Even for such an adiabatic

trajectory, we get survival probability nearly equal to that obtained with CDF, although our method yields slightly higher values (see Fig. S-2 B, black and red curves). To find a non-adiabatic optimal trajectory for this parameter regime, for fast transport of wavepackets, we can tune λ, which controls the total energy input for the transport. We find that for $\lambda = 0.07$ the optimal trajectory is non-adiabatic (Fig. S-2 A, green curve) with $v(t_f) = 0$ and the corresponding survival probabilities are much higher in our method than in CDF, both in the presence and in the absence of a bath (Fig. S-2 B, C). This result confirms the optimality of our method over a wide range of parameter values.

The derived optimal velocity profiles all satisfy the condition $|\dot{x}_\circ(t)| = |v(t)| < v_s = \big|(\omega_{\epsilon n} + \Omega_k)/k\big|$ necessary for the validity of the FDA and hence of the linearized Eq. (8) of the manuscript. To see this we compare the maximum value of the derived optimal velocity $|v(t)|_{\max}$ with the minimum value of v_s i.e. $v_s|_{\min}$. We note that v_s is a function of k and ϵ while $|v(t)|_{\max}$ is independent of k, ϵ. From Fig. (S-3) we conclude that

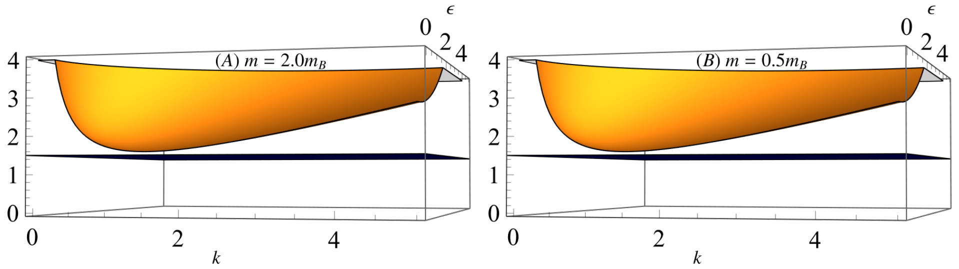

Supplementary Fig. S-3 Validity of the linearized Eq. (8): A Plot of v_s and $|v(t)|_{\max}$ for $m = 2.0\,m_B$ and $\tilde{g} = 1.0\,t_B^{-1}$. The yellow surface shows v_s while the plane (black) indicates $|v(t)|_{\max}$. **B** Same for $m = 0.5\,m_B$. All other parameters are as described in Methods C.

$$|v(t)|_{\max} < v_s|_{\min} \tag{S78}$$

and hence

$$|\dot{x}_\circ(t)| = |v(t)| < v_s\ \forall\ k,\ \epsilon,\ t. \tag{S79}$$

Importantly, we have shown in Fig. S-1 that a numerical solution of the full non-linear integro-differential equation, Eq. (12) together with Eq. (13), with appropriate BCs using successive Picard iterations, converge to the solutions obtained from the linearized Eq. (8) derived using FDA. This result itself confirms the validity of the FDA and hence the condition $|v(t)| < v_s$ in our results.

XII Equivalence of different boundary conditions: Uniqueness of the optimal trajectory

In Sec. X we have presented the Green's function for the BCs $v(0) = 0$ and $v(t_f) = 0$. Alternatively, we here impose BCs on $x(0), v(0)$, and $x(t_f)$ and explicitly show how appropriate choices of these quantities yield identical velocity profiles, $v(t)$ (and hence identical time dependence of survival probabilities) for the two types of BCs.

As $\dot{x}_\circ(t) = v(t)$, Eq. (S65) can be written as

$$\dddot{x}_\circ(t) = \frac{1}{\lambda}\mathcal{M}(t) \equiv g(t). \tag{S80}$$

We solve the above equation under the BCs: $x_\circ(0) = 0, v(0) = 0$, and $x_\circ(t_f) = x_f$. The Green's function for a third order differential equation with the given boundary conditions is obtained as follows [s38]. Integrating Eq. (S80) with respect to t we get

$$\ddot{x}_\circ(t) = \dot{v}(t) = d_2 + \int_0^t g(s)ds. \tag{S81}$$

where d_2 is a constant, to be determined by the BCs. Subsequent integrations over t yields

$$\dot{x}_\circ(t) = v(t) = d_1 + d_2 t + \int_0^t (t-s)g(s)ds. \tag{S82}$$

$$x_\circ(t) = d_0 + d_1 t + \frac{1}{2}d_2 t^2 + \frac{1}{2}\int_0^t (t-s)^2 g(s)ds. \tag{S83}$$

where d_0 and d_1 are similar constants determined by the BCs. Imposing the BCs $x_\circ(0) = 0, v(0) = 0$, and $x_\circ(t_f) = x_f$ we then get

$$d_0 = 0, d_1 = 0, \text{ and } d_2 = \frac{2x_f}{t_f^2} - \frac{1}{t_f^2}\int_0^{t_f} (t_f - s)^2 g(s)ds$$

which yields

$$v(t) = \frac{2x_f t}{t_f^2} - \frac{t}{t_f^2}\int_0^{t_f} (t_f - s)^2 g(s)ds + \int_0^t (t-s)g(s)ds.$$

$$\implies v(t) = \frac{2x_f t}{t_f^2} + \int_0^t \left[(t-s) - \frac{t}{t_f^2}(t_f - s)^2\right] g(s)ds + \int_t^{t_f} \left[-\left(\frac{t}{t_f^2}\right)(t_f - s)^2\right] g(s)ds. \tag{S84}$$

Thus, the Green's function for the problem is

$$G(t,s) = \begin{cases} \left[(t-s) - \frac{t}{t_f^2}(t_f - s)^2\right] & 0 \le s \le t \le t_f \\ \\ \left[-\left(\frac{t}{t_f^2}\right)(t_f - s)^2\right] & 0 \le t \le s \le t_f. \end{cases} \tag{S85}$$

and the corresponding optimal velocity profile is given by

$$v(t) = \frac{2x_f t}{t_f^2} + \int_0^{t_f} ds\, G(t,s)\, g(s). \tag{S86}$$

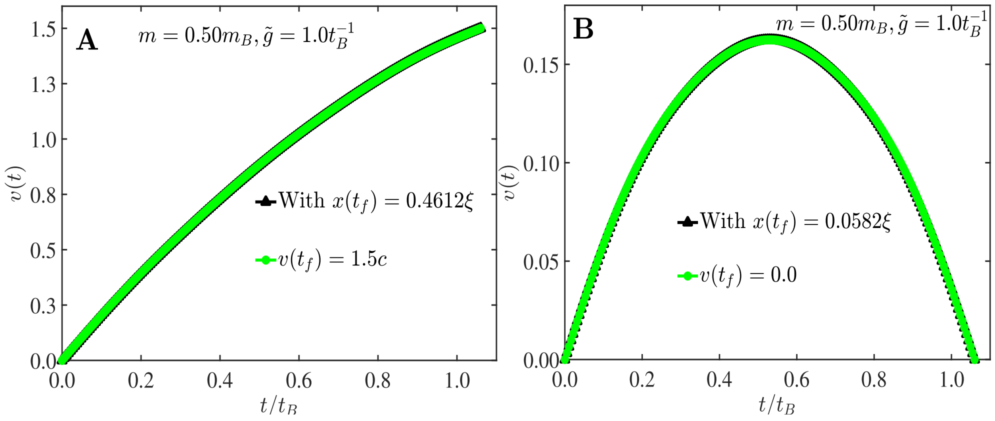

Supplementary Fig. S-4 Comparison of the velocity profile $v(t)$ obtained from two boundary conditions: A The profile $v(t)$ obtained for $v(0) = 0$ and $v(t_f) = 1.5c$ matches exactly with $v(t)$ obtained for $x_\circ(0) = 0, v(0) = 0$, and $x_\circ(t_f) = x_f = \int_0^{t_f} v(t)dt = 0.4612\,\xi$. **B** Same for $v(0) = 0$ and $v(t_f) = 0$ with $x_\circ(t_f) = x_f = 0.0582\,\xi$.

Eq. (S86) can be written in the form of Eq. (S69) i.e. a Fredholm integral equation of second kind in v, by absorbing the term $\frac{2x_f t}{t_f^2}$ in the inhomogeneous part $\mathcal{F}(t)$. Then Eq. (S86) can be solved directly following the method described in Sec. X.

To compare the survival probabilities obtained for a given t_f under the BCs: (i) $v(0) = 0$,$v(t_f) = c_1 \neq 0$, $x_\circ(0) = 0$ and (ii) $x_\circ(0) = 0, v(0) = 0$, $x_\circ(t_f) = x_f \neq 0$, we first find the optimal velocity $v^{(i)}(t)$ corresponding to (i) and evaluate the corresponding $x_\circ^{(i)}(t_f)$ as the area under the velocity profile. We then find the optimal velocity $v^{(ii)}(t)$ corresponding to BCs (ii) for the same final trap centre position $x_\circ^{(i)}(t_f)$ i.e. by setting $x_f = x_\circ^{(i)}(t_f)$. In Fig. S-4 we explicitly show that both these BCs yield identical velocity profiles $v^{(i)}(t)$ and $v^{(ii)}(t)$ and hence the corresponding optimal transport tajectories $x_\circ(t)$ given by the area under the velocity curves and survival probabilities $\mathcal{P}(t_f)$ given by (S34–S35) are identical. We thus conclude that the BCs (i) and (ii) yield equivalent results confirming the uniqueness of the derived optimal trajectory as explained in Sec. X.

XIII Solution of the nonlinear problem using Picard method of successive approximations

We have implemented a Picard iteration scheme to solve the full non-linear integro-differential equation, without invoking the Frequency Discrimantor Approximation (FDA) (see text). Using the Picard method, we wish to solve a two-point BVP of the form

$$\ddot{v}(t) = f(t, v, \dot{v}) \tag{S87}$$

with BCs $v(t_0) = \alpha$ and $v(t_f) = \beta$ along with (S62) with $x_\circ(0) = 0$. Here, t_0 and t_f represent the initial and the final boundary points respectively. From the Picard-Lindelof theorem, we know that if f is a locally Lipschitz function v and $\dot{v}$, then for any $\gamma \in R$ the initial value

problem

$$\ddot{v}(t) = f(t, v, \dot{v}); v(t_0) = \alpha, \dot{v}(t_0) = \gamma \tag{S88}$$

has a unique solution in an interval about $t = t_0$. Introducing the variable $u = \dot{v}$ we obtain the equivalent set of first order differential equations:

$$\dot{v} = u \tag{S89}$$
$$\dot{u} = f(t, v, u) \tag{S90}$$
$$v(t_0) = \alpha, u(t_0) = \gamma. \tag{S91}$$

We can express (S88) in the equivalent integral form

$$v(t) = \alpha + \gamma(t - t_0) + \int_{t_0}^{t} (t - s) f(s, v(s), \dot{v}(s)) ds \tag{S92}$$

which we then solve for γ using the BC, $v(t_f) = \beta$

$$\gamma = \frac{1}{t_f - t_0} \left(\beta - \alpha - \int_{t_0}^{t_f} (t_f - s) f(s, v(s), \dot{v}(s)) ds \right). \tag{S93}$$

We next use Picard iterations to obtain successive approximations to the value of γ as:

$$v^{(0)}(t) = \alpha \tag{S94}$$
$$u^{(0)}(t) = \frac{\beta - \alpha}{t_f - t_0} \tag{S95}$$
$$\gamma^{(0)}(t) = \frac{\beta - \alpha}{t_f - t_0} \tag{S96}$$
$$u^{(n+1)}(t) = \alpha + \int_{t_0}^{t_f} u^{(n)}(s) ds \tag{S97}$$
$$v^{(n+1)}(t) = \gamma^{(n)} + \int_{t_0}^{t_f} f(s, v^{(n)}, u^{(n)}(s)) ds \tag{S98}$$
$$\gamma^{(n+1)} = \frac{1}{t_f - t_0} \left(\beta - \alpha - \int_{t_0}^{t_f} (t_f - s) f(s, p^{(n)}, u^{(n)}(s)) ds \right) \tag{S99}$$

with $n = 1, 2, 3 \cdots$. In our case,

$$f(t, v, \dot{v}) = \frac{1}{\lambda} \left[-\eta_1(t) - \zeta_1(t) v(t) + \int_0^{t_f} ds\, \phi(t - s)\, v(s) \right]$$

with the following defining equations for $\eta_1(t), \zeta_1(t)$

$$\eta_1(t) = \int_0^{t_f} ds\, \frac{L}{2\pi} \int d\epsilon\, dk\, k\, (\omega_{\epsilon n} + \Omega_k)\, |g_k\, d^k_{n\epsilon}|^2\, \cos\Big[(\omega_{\epsilon n} + \Omega_k)(t-s) + k\{x_\circ(t) - x_\circ(s)\}\Big],$$

$$\zeta_1(t) = \int_0^{t_f} ds\, \frac{L}{2\pi} \int d\epsilon\, dk\, k^2\, |g_k\, d^k_{n\epsilon}|^2 \cos\Big[(\omega_{\epsilon n} + \Omega_k)(t-s) + k\{x_\circ(t) - x_\circ(s)\}\Big]. \qquad \text{(S100)}$$

Supplementary References

[s1] Morse, P. M. Diatomic molecules according to the wave mechanics. II. Vibrational levels. *Phys. Rev.* **34**, 57 (1929).

[s2] de Lima, E. F. & Hornos, J. E. M. Matrix elements for the Morse potential under an external field. *J. Phys. B: At. Mol. Opt. Phys.* **38**, 815 – 825 (2005).

[s3] de Lima, E. F. & Hornos, J. E. M. The Morse oscillator under time-dependent external fields. *J. Chem. Phys.* **125**, 164110 (2006).

[s4] Leonard, A. & Deffner, S. Quantum work distribution for a driven diatomic molecule. *Chem. Phys.* **446**, 18 (2015).

[s5] Kofman, A. G. & Kurizki, G. Universal dynamical control of quantum mechanical decay: modulation of the coupling to the continuum. *Phys. Rev. Lett.* **87**, 270405 (2001).

[s6] Fröhlich, H. Electrons in lattice fields. *Adv. Phys.* **3**, 325–361 (1954).

[s7] Kolodrubetz, M., Sels, D., Mehta, P. & Polkovnikov, A. Geometry and non-adiabatic response in quantum and classical systems. *Phys. Rep.* **697**, 1 – 87 (2017).

[s8] Sakurai, J. J. & Fu Tuan, S. *Modern Quantum Mechanics* (Addison-Wesley Publishing Company, Reading, 1994).

[s9] Beauchard, K. & Coron, J-M. Controllability of a quantum particle in a moving potential well. *J. Funct. Anal.* **232**, 328 (2006).

[s10] Rouchon, P. Control of a quantum particle in a moving potential well. *IFAC Proc. Vol. (IFAC-PapersOnline)*, **36**, 287–290 (2003).

[s11] Sels, D. & Polkovnikov, A. Minimizing irreversible losses in quantum systems by local counterdiabatic driving. *Proc. Natl. Acad. Sci. U.S.A.* **114**, E3909–E3916 (2017).

[s12] Maamache, M. & Saadi, Y. Adiabatic theorem and generalized geometrical phase in the case of continuous spectra. *Phys. Rev. Lett.* **101**, 150407 (2008).

[s13] Maamache, M. & Saadi, Y. Quantal phase factors accompanying adiabatic changes in

the case of continuous spectra. *Phys. Rev. A* **78**, 052109 (2008).

[s14] Cohen-Tannoudji, C., Diu, B. & Laloe, F. *Quantum Mechanics* Vol. II Ed. 2 (Wiley-VCH Verlag, Weinheim, 2020).

[s15] Cohen-Tannoudji, C., Dupont-Roc, J. & Grynberg, G. *Atom-photon Interactions: Basic Processes and Applications* (Wiley-VCH Verlag, Weinheim, 2004).

[s16] Breuer, H-P. & Petruccione, F. *The Theory of Open Quantum Systems* (Oxford university press, Oxford, 2002).

[s17] Keitel, C. H., Knight, P. L., Narducci, L. M. & Scully, M. O. Resonance fluorescence in a tailored vacuum. *Opt. Commun.* **118**, 143–153 (1995).

[s18] Riera-Campeny, A., Sanpera, A. & Strasberg, P. Quantum systems correlated with a finite bath: nonequilibrium dynamics and thermodynamics. *PRX Quantum* **2**, 010340 (2021).

[s19] Nielsen, K. K., Ardila, L. A. P., Bruun, G. M. & Pohl, T. Critical slowdown of non-equilibrium polaron dynamics. *New J. Phys.* **21**, 043014 (2019).

[s20] Gordon, G., Erez, N. & Kurizki, G. Universal dynamical decoherence control of noisy single-and multi-qubit systems. *J. Phys. B: At. Mol. Opt. Phys.* **40**, S75 (2007).

[s21] Khalfin, L. A. Contribution to the decay theory of a quasi-stationary state. *Sov. Phys. JETP* **6**, 1053–1063 (1958).

[s22] Kofman, A. G. & Kurizki, G. Acceleration of quantum decay processes by frequent observations. *Nature* **405**, 546–550 (2000).

[s23] Boyanovsky, D. & Holman, R. On the perturbative stability of quantum field theories in de Sitter space. *JHEP* **2011**, 1–37 (2011).

[s24] Boyanovsky, D., Jasnow, D., Wu, X-L. & Coalson, R. C. Dynamics of relaxation and dressing of a quenched Bose polaron. *Phys. Rev. A* **100**, 043617 (2019).

[s25] Scully, M. O. and Zubairy, M. S. *Quantum Optics* (Cambridge University Press, Cambridge, 1997).

[s26] Sargent, M. III, Scully, M. & Lamb, W. E. *Laser Physics* (Addison-Wesley, Reading, 1974).

[s27] Louisell, W. H. *Quantum Statistical Properties of Radiation* (John Wiley and Sons, Inc., New York, 1990).

[s28] Nielsen, M. A. and Chuang, I. L. *Quantum Computation and Quantum Information* (Cambridge University Press, Cambridge, 2010).

[s29] Liang, Y-C., Yeh, Y-H., Mendonça, P. E. M. F., Teh, R. Y., Reid, M. D. & Drummond,

P. D. Quantum fidelity measures for mixed states. *Rep. Prog. Phys.* **82**, 076001 (2019).

[s30] Mendonça, P. E. M. F., Napolitano, R. d. J., Marchiolli, M. A., Foster, C. J., Liang, Y-C. Alternative fidelity measure between quantum states. *Phys. Rev. A* **78**, 052330 (2008).

[s31] Schumacher, B. Quantum coding. *Phys. Rev. A* **51**, 2738–2747 (1995).

[s32] Edelen, D. G. B. Non-local variational mechanics – I stationarity conditions with one unknown. *Int. J. Engng. Sci.* **7**, 269 – 285 (1969).

[s33] Abramowitz, M. & Stegun, I. A. *Handbook of Mathematical Functions with Formulas, Graphs, and Mathematical Tables* Tenth Printing, Ch. 13, 504 – 505 (National Bureau of Standards, Applied Mathematics Series - 55, Washington, D.C., 1972).

[s34] Dixit, A. & Moll, V. H. The integrals in Gradshteyn and Ryzhik Part 28: The confluent hypergeometric function and Whittaker functions. *Scientia, Ser. A: Math. Sci.* **26**, 49 – 61 (2015).

[s35] Tamarkin, J. D. The notion of the Green's function in the theory of integro-differential equations. *Trans. Am. Math. Soc.* **29**, 755–800 (1927).

[s36] Singh, R. & Wazwaz, A-M. Numerical solutions of fourth-order Volterra integro-differential equations by the Green's function and decomposition method. *Math. Sci.* **10**, 159–166 (2016).

[s37] Arfken, G. B., Weber, H. J. & Harris, F. E. *Mathematical Methods for Physicists A Comprehensive Guide Ed. 7.* (Elsevier, Academic Press, Waltham, 2013).

[s38] Morrison, S. M. *Application of the Green's Function for Solutions of Third Order Nonlinear Boundary Value Problems* (Master's Thesis, University of Tennessee, https://trace.tennessee.edu/utk_gradthes/174, Knoxville, 2007).
